# Quantifying national burdens of foodborne disease—Four imperatives for global impact

**DOI:** 10.1371/journal.pgph.0004309

**Published:** 2025-04-09

**Authors:** Karen H. Keddy, Sandra Hoffmann, Luria Leslie Founou, Teresa Estrada-Garcia, Tesfaye Gobena, Arie H. Havelaar, Lea Sletting Jakobsen, Kunihiro Kubota, Charlee Law, Rob Lake, Yuki Minato, Fadi Nasr Radwan Al-Natour, Sara M. Pires, Tety Rachmawati, Banchob Sripa, Paul Torgerson, Elaine Scallan Walter

**Affiliations:** 1 Department of Veterinary Tropical Diseases, Faculty of Veterinary Science, University of Pretoria, Pretoria, South Africa; 2 US Department of Agriculture, Economic Research Service, Washington, DC, United States of America; 3 Reproductive, Maternal, Newborn, and Child Health (ReMARCH) Research Unit, Research Institute of the Centre of Expertise and Biological Diagnostic of Cameroon (CEDBCAM-RI), Yaoundé, Cameroon; 4 Antimicrobial Research Unit, School of Health Sciences, College of Health Sciences, University of KwaZulu-Natal, Durban, South Africa; 5 Infection and Global Health Division, School of Medicine, University of St Andrews, St Andrews, Scotland; 6 Biomedicine Department of the Center for Research and Advanced Studies of the National Polytechnic Institute (CINVESTAV-IPN), Mexico City, Mexico; 7 College of Health and Medical Science, Haramaya University, Harar, Ethiopia; 8 Department of Animal Sciences, Global Food Systems Institute, and Emerging Pathogens Institute, University of Florida, Gainesville, Florida, United States of America; 9 National Food Institute, Technical University of Denmark, Kongens Lyngby, Denmark; 10 Division of Food Safety information, National Institute of Health Sciences, Kawasaki, Japan; 11 Monitoring and Surveillance Nutrition and Food Safety Unit, Department of Nutrition and Food Safety, World Health Organization, Geneva, Switzerland; 12 Institute of Environmental, Science and Research, Auckland, New Zealand; 13 Abu Dhabi Agriculture and Food Safety Authority, Abu Dhabi, United Arab Emirates; 14 Center of Research and Development for Humanities and Health Management, Jakarta, Indonesia; 15 Tropical Disease Research Center, Faculty of Medicine, Khon Kaen University, Khon Kaen, Thailand; 16 Veterinary Epidemiology, University of Zürich, Zürich, Switzerland; 17 Department of Epidemiology, Colorado School of Public Health, Aurora, Colorado, United States of America; ETH Zürich: Eidgenossische Technische Hochschule Zurich, SWITZERLAND

## Abstract

Estimates of national burdens of the foodborne disease (FBD) inform country-level food safety policies, ranking infectious and non-infectious FBD hazards in terms of health and socioeconomic impact to mitigate FBD burdens. Using relevant publications on FBD burdens from scientific literature, this review contends that four major imperatives (health, economic, planetary boundaries, governance) argue for a sustainable programme to quantify national FBD burdens. FBD disproportionately affects children under five years of age, and low- and middle-income countries. The economic costs are significant and include medical care, child development, lost productivity and international trade losses. Climatic changes and environmental contamination cause socio-ecological disruptions, increasing risk factors for FBD. Good governance promotes food safety initiatives, addressing in part under-diagnosis and underreporting. Strengthening national policies on FBD surveillance and burden estimation can promote food safety policies and address the global and national imperatives for FBD control. Evidence-based educational and regulatory interventions for FBD can promote improvements in the health and socioeconomic circumstances of the most vulnerable.

## Introduction

The first global assessment of the burden of foodborne disease (FBD), conducted by the World Health Organization (WHO) Foodborne Disease Burden Epidemiology Reference Group (FERG) for 2007–2015 [1], estimated 31 foodborne agents caused 600 million (95% uncertainty interval [UI] 420–960) foodborne illnesses in 2010, and 420,000 (95% UI 310,000–600,000) deaths [2]. WHO FERG estimated FBD acute illness and death, and chronic illness morbidity and mortality caused 33 (95% UI 25–46) million Disability Adjusted Life Years (DALYs) in 2010, an overall burden of disease (BoD) comparable with major infectious diseases of HIV/AIDS, malaria, or tuberculosis [3]. Limited data on potentially important foodborne agents and disease sequelae in many WHO Member States, where high FBD burden are expected, renders these WHO estimates a conservative reflection of the true human health impact of FBD.

Exploiting the full potential of BoD estimates can enhance national food safety policies by ranking FBD in terms of the health and economic impacts on the population. Specifically, the foods which contribute significantly to disease burdens can be identified and targeted for interventions in the food chain. This requires effective reporting, knowledge transfer and communication at national and subnational levels. Outputs from the burden of FBD studies are also essential to identify further research requirements and data collection, raising awareness for food safety issues in the general population – among citizens, health stakeholders, industry, and educators alike. The review is intended to provide a comprehensive, evidence-based narrative for policymakers, researchers and funders to understand the extent that FBD can affect health and wellbeing, and so encourage countries to increase their capacity to collect surveillance data and assess the burden of FBD. The review is set in the context of the current WHO work to update the FBD estimates published in 2015.

## Methods and rationale

This narrative review examines four major global and national imperatives for development of standardised and sustained FBD surveillance and burden estimation programme at a national level: health effects, economic costs, the effect of exceeding planetary boundaries, and governance for improved data and tools to better manage food safety. These imperatives were guided by the structure of the current FERG (FERG2), which includes taskforces for burden estimations of foodborne health hazards (Enteric, Parasitic, and Chemical and Toxins Task Forces, Source Attribution Task Force, and Computational Task Force [EDTF, PDTF, CTTF, SATF, CTF] respectively) 2021–2025) (https://cdn.who.int/media/docs/default-source/foodborne-diseases/ferg/ferg-task-force-structure-and-members.pdf) [4]. The impact of these hazards on global populations, including public health and economic burden, overextension of planetary boundaries changing disease burdens is discussed. Addressing foodborne hazards could curtail FBD BoD (Impact Measurement Task Force [IMTF] and Country Support Task Force [CSTF], supporting the WHO Global Strategy for Food Safety 2022–2030) [4,5].

Quantifying national FBD burdens enables countries to use these data to formulate risk ranking of different foodborne hazards, providing an evidence base to design appropriate interventions to mitigate disease burdens and sequelae. Introduction of formal national policies to create an iterative process for these activities will enable measurement of health and socioeconomic impacts of any interventions on national populations, while enabling earlier and enhanced identification of emerging hazards and FBD outbreaks.

### Search strategy and selection criteria

We identified peer-reviewed publications on PubMed, Google Scholar addressing FBD burdens, and accessed official national and United Nations (WHO, FAO, World Bank) reports examining FBD burdens, using search terms including foodborne, hazard groups as defined above [4] and individual hazards (*Salmonella, Taenia solium*, lead etc.), burden of disease or BoD, sequelae, economic, climate change, planetary boundaries, and governance, amongst others. Further illustrative publications were identified through references in the text of these manuscripts.

Pertinent publications were identified between February 2022 and January 2025 that described FBD burdens at a national or supranational level. Publications were screened by title by the subject matter authors of this manuscript, and then abstracts were reviewed for relevance. As this was not intended as a systematic review, a structured database of those articles identified and included or excluded was not maintained.

### Ethics

To our knowledge, none of the information or data included here has been identified as having ethically unsound methodology or flagged as fraudulent.

### Illustrative examples

Publications reporting FBD data identified through searching the literature were used to create Table 1. This Table provides examples of foodborne disease estimates and potential changes in relation to the health, economic and planetary boundary imperatives identified above. Governance aspects are discussed in a later section.

**Table 1:** Selected hazard-specific examples of foodborne disease, estimated costs of disease, and modelled changes in disease patterns due to extreme weather events, integrating the health, economic and climate (planetary boundary) imperatives, where data were available.

Foodborne hazard	Estimated foodborne illness burden of disease in 2010 (% foodborne)	Estimated costs^b^	Estimated changes in burden of foodborne disease and/or mortality due to climate change
*Campylobacter*	96 million cases (58%) and contributing to greater proportions of disability adjusted life years (DALYs) in high-income countries (HICs). [24]	USD 1,846 productivity and USD 8,141 quality adjusted life years (QALYs) per case in the United States in 2010 [30].	Temperature increases will increase incidence of *Campylobacter* foodborne illnesses will increase between 2 and 3% in Ireland due to climate change [31].
		GBP 2,400 per case in 2018 in the United Kingdom [32].	Increases in temperature and precipitation will double the burden of *Campylobacter* cases with an additional 6000 cases/year in Denmark, Finland, Norway, and Sweden by 2080 [33].
		EURO 757 per case in the Netherlands in 2011 [34].	An increase of the order of 4.5°C in average temperature forecast by 2055 resulting in ^a^ 23% increase in incidence of *Campylobacter* cases equivalent to 4,000 extra cases per year in Montreal, Canada [35].
		AUD 1383 total cost per case in Australia in 2019 [36].	The mean attributable fraction of deaths due to climate change will increase from 2.15 between 2020 and 2035 to 5.79 from 2080 to 2095^c^ [37].
			NZD 872 total cost per case in 2009 in New Zealand [38].
*Salmonella enterica* serotype Typhi	7 million cases (37%) and associated with excessive DALYs in low middle-income countries (LMICs) [24].	USD 4,293 productivity and USD 11,488 QALYs per case in the United States in 2010 [30].	Increases in temperature will result in 4000–7000 additional cases in Australia by 2050 [39].
		AUD 16,207 total cost per case in Australia in 2019 [36].	The mean attributable fraction of deaths due to climate change will increase from 12.64 between 2020 and 2035 to 31.52 from 2080 to 2095.^c^ [37]
Non-invasive nontyphoidal *Salmonella*	78 million cases (52%) and not geographically restricted to HICs or LMICs [24].	USD 4,312 productivity and USD 11,086 QALYs per case in the United States in 2010 [30].	Incidence of *Salmonella* foodborne illnesses will increase between 2 and 3% in Ireland [31].
		GBP 6,700 per case in 2018 in the United Kingdom [32].	The mean attributable fraction of deaths due to climate change will increase from 4.91 between 2020 and 2035 to 12.87 from 2080 to 2095.^c^ [37]
			EURO 640 per case in the Netherlands in 2011 [34].
			AUD 2,272 per case in Australia in 2019 [36].
			NZD 5,622 per case in New Zealand in 2009 [38].
Enteropathogenic *E. coli*	24 million cases (30%) and associated with excessive DALYs in LMICs [24].		The mean attributable fraction of deaths due to climate change will increase from 3.94 between 2020 and 2035 to 10.49 from 2080 to 2095.^c^ [37]
Norovirus	125 million cases (18%) and contributes to ^a^ greater proportion of DALYs in HICs [24].	USD 530 productivity and USD 633 QALYs per case in the United States in 2010 [30].	Extreme temperatures may cause increases then normalization between 2030 and 2100, due to ^a^ winter predominance in transmission.^d^ [40–42]
		GBP 4,400 per case in 2018 in the United Kingdom [32].	The mean attributable fraction of deaths due to climate change will decrease from -12.8 between 2020 and 2035 to -39.09 from 2080 to 2095.^c^ [37]
			EURO 152 per case in the Netherlands in 2011 [34].
			AUD 390 per case in Australia in 2019 [36].
			NZD 362 total costs per case in New Zealnd in 2009 [38].
*Shigella*	51 million cases (27%) and associated with excessive DALYs in LMICs [24].	GBP 7,500 per case in 2018 in the United Kingdom [32].	Increased disease burdens between 11 and 20% in China are expected by 2090 [43].
		AUD 1,767 per case in Australia in 2019 [36].	The mean attributable fraction of deaths due to climate change will increase from 6.44 between 2020 and 2035 to 16.7 from 2080 to 2095.^c^ [37]
Enterotoxigenic *E. coli*	87 million cases (36%) and associated with excessive DALYs in LMICs. [24]	USD 863 productivity and USD 1,334 QALYs per case in the United States in 2010 [30].	Increased relative risk (RR)=1.73 during floods due to La Niña in Peru 2011 – 2012 [44].
			Significantly increased burdens were described with increased temperature (RR=1.40) in MAL-ED [45].
			The mean attributable fraction of deaths due to climate change will increase from 3.97 between 2020 and 2035 to 10.51 in 2080–2095.^c^ [37]
Enterohaemorrhagic *E. coli*	1,2 million cases (48%) and contributes to ^a^ greater proportion of DALYs in HICs [24].	USD 9,606 productivity and USD 10,048 QALYs per case in the United States in 2010 [30].	EHEC will increase by 10% in Ireland [37].
		GBP 8,400 per case in 2018 in the United Kingdom [32].	Water-limited resources will have an impact on human health and FBD as well; droughts increase the use of sewage water for irrigation, including plants that are consumed raw as spinach, lettuce etc. So far, has been demonstrated that EHEC 0157:H7 penetrates through spinach leaves stomas and then multiplies inside the plant tissue, making impossible its elimination by household traditional disinfectants [46].
			EURO 4,668 in the Netherlands in 2011 [34].
			AUD 4,449 per case in Australia in 2019 [36].
			NZD 69,667 total cost per case in New Zealand in 2009 [38].
*Vibrio parahaemolyticus*	92,402 cases (United States, 2018) (~80%) [47].	USD 1,957 productivity and USD 2,551 QALYs per case in the United States in 2010 [30].	At RCP4.5, the anticipated increase in the total number of cases will be 134,454 by 2050 and 151,402 by 2090, compared with 147,294 (2050) and 221,177 (2090) at RCP8.5 [47].
Aflatoxins	21,757 cases (100%) and associated with excessive DALYs in LMICs [1].		During extreme drought conditions in Serbia in 2012, aflatoxins were identified in 72% of maize samples, compared with 5% of samples in moderate weather conditions in 2016 [48].
			Aflatoxin B1 is predicted to become ^a^ food safety issue in maize in Europe, if there is ^a^ 2°C or greater rise in temperature [49].
Mercury	Consumption of methyl mercury has resulted in between 1.5/1000 and 17/1000 children in selected fishing populations having cognitive impacts [23].		Methyl mercury concentration in fish increased 20% under drought conditions in Brazil in 2014 [50].
Dioxins	193,447 cases [1]; (>90% due to foodborne exposure) [51].		Concentrations of some dioxins increased in freshwater fish in Germany downstream from industrial sites between 1995 and 2014 [52].

Due to differences in data collection methodologies and absence of data from certain regions, the data among the different hazards may not all be comparable.

^a^Further information is included in the discussions in the sections on economic, planetary boundary and governance imperatives.

^b^Data are only available for certain countries and there are country-specific as well as time-bound differences in how these were reported. Total costs in the United States were estimated at USD 51 billion (2010$ USD values) direct and indirect costs annually [30], at GBP 9.1 billion (2018 GBP) in the United Kingdom [32], AUD 2.4 billion (2019 AUD) in Australia [36], EURO 423–467 million (2019 Euros) in the Netherlands [53] and NZD 162 million for the six major pathogens in New Zealand including those tabled above, listeriosis and yersiniosis (2009 NZD) [38].

^c^If there is limited national investment in health strategies and insufficient action is taken to limit greenhouse gas emissions calculated at a Representative Concentration Pathway (RCP) of 6, one of the trajectories defined by the Intergovernmental Panel on Climate Change (IPCC) for greenhouse gas concentrations in years to come, with emissions peaking in 2080 [37].

^d^Modelling does not replace measurement – unforeseen circumstances may affect disease burdens, underscoring the need for well-conducted sustained national surveillance for foodborne disease and burden of disease studies. During Hurricane Katrina in the United States in 2005, over 1000 patients from a “megashelter” were treated for Norovirus [42]. Conversely, during the COVID-19 pandemic, Norovirus cases reportedly decreased by 49% in both United States and Australia [40,41].

## The health imperative

Foodborne diseases include a broad group of illnesses caused by consuming contaminated foods including infectious and non-infectious hazards, comprising a multitude of bacteria, viruses, parasites, and chemicals. The spectrum of FBD is constantly changing with hazards not previously considered foodborne found to be so, including *Cronobacter sakazakii* [6] in powdered infant formula and, in South America, *Trypanosoma cruzi*, causing Chagas disease [7,8], and cancer due to dietary expose to acrylamide [9].In addition, some foodborne pathogens are developing resistance to antimicrobial drugs [10–17], complicating treatment options, worsening clinical outcomes, prolonging hospital stays, and increasing medical care costs (see section on the economic imperative).

### Long-term complications and chronic sequelae

FBD is often associated with mild gastrointestinal symptoms, including diarrhoea and vomiting, however, more severe gastrointestinal, neurological and other diseases including cancers, resulting in acute hospitalization, long-term disability and death are well described. Long-term complications and chronic sequelae include impaired kidney function after Shiga toxin-producing *Escherichia coli* (STEC) infection [18], Guillain-Barré syndrome after *Campylobacter* infection [19], hearing and vision impairment after listeriosis and toxoplasmosis, hepatocellular carcinoma in association with aflatoxins [1], and reactive arthritis, irritable bowel syndrome, epilepsy, and other neurological sequelae after a variety of FB infections [20]. Dietary chemical exposures are also associated with a range of chronic, multi-causal diseases including lead-induced high systolic blood pressure leading to cardiovascular disease (CVD) [21] and hepatocellular carcinoma caused by aflatoxin [22]. CVD and liver cancer are diseases of considerable public health impact, and even if the proportion of the disease ascribed to food is limited, it may still pose a high burden. Other adverse health effects caused by chemical exposures through food include dioxin-induced infertility [22], cadmium-induced chronic kidney disease and intellectual disability in children caused by maternal exposure to methyl mercury [23].

Incubation times for the health effects of FBD caused by bacterial hazards range from hours to weeks, while the effects of certain parasite eggs may take years to develop. Chronic exposure to hazardous chemicals in foods over decades may be required for adverse health effects. Thus, identifying causal relationships and measuring the long-term health impacts of FBD are difficult. Further complicating estimation, chronic diseases such as cancer, kidney or liver failure are often multi-causal, and require delineation of foodborne exposures from other sources. Likewise, the substantial contribution of FBD to foetal loss and stillbirths, and the long-term health impact on people with immunocompromising conditions, including HIV/AIDS, is often underestimated. Of note, the burden of FBD on individuals with HIV/AIDS were not included in the 2010 estimates. This avoided double-counting in WHO estimates but certainly underestimated the burden of FBD.

### High burden in young children and low- and middle-income countries

Those at greater risk of acquiring FBD include young children, pregnant women and their unborn babies, older adults, and people with weakened immune systems, such as those with HIV/AIDS, cancer, diabetes, kidney disease, or transplant patients. WHO estimated that children under five years of age suffered 40% of the estimated disease burden despite only representing 9% of the global population [3]. The greatest burden is in sub-Saharan Africa (SSA) and Southeast Asia (SEA) where children under five years bear 63% of the FBD burden of infection and 50% of the FBD-associated mortality [24]. This is unsurprising given that diarrhoea is a leading cause of death worldwide among children under five years of age and much of the FBD burden in children is due to diarrhoeal disease agents. Furthermore, invasive salmonellosis is a more frequent complication in sub-Saharan Africa (SSA) than in other regions and is associated with a high case-fatality ratio [24].

Low- and middle-income countries (LMICs) are disproportionately affected by FBD. Of the 14 subregions included in the WHO assessment, the largest disease burden was seen in the SSA, followed by those of South-East Asia (SEA) and the Eastern Mediterranean. For impoverished children living in low-resource settings, diarrhoeal disease agents transmitted through food likely have a substantial impact on malnutrition, with an estimated 161.8 million malnourished children <5 years affected, and an excessive burden of foodborne illness in SSA in 2019 [25–28].

### Leading causes of foodborne disease globally and regionally

**Fig 1:**
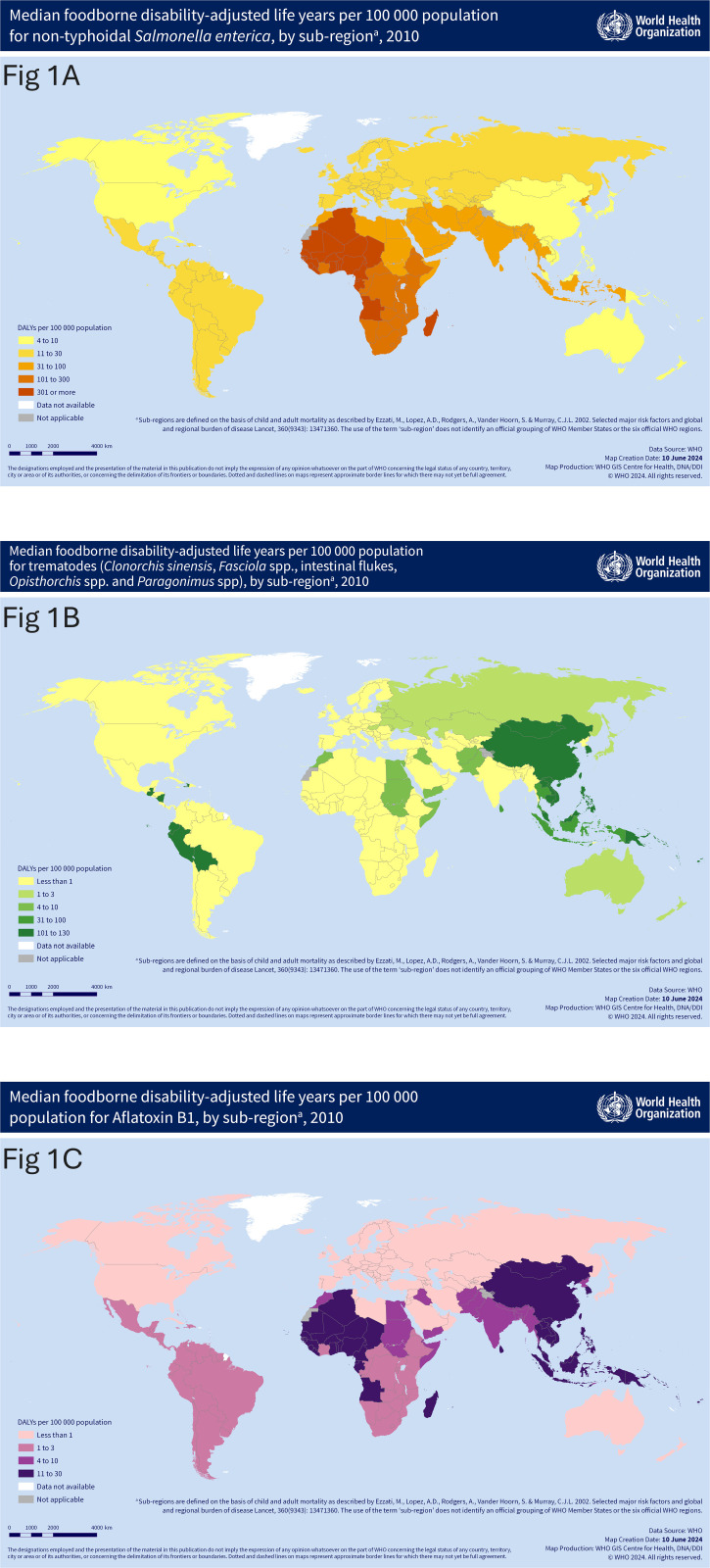
Median subregional differences for foodborne disability-adjusted life years (DALYs) per 100 000 population associated with (A) Non-typhoidal *Salmonella enterica* ( https://www.who.int/images/default-source/maps/ferg2010_non-typhoidal_s__enterica_a3_20240610.png), (B) Trematodes (*Clonorchis sinensis*, *Fasciola* spp., Intestinal flukes, *Opisthorchis* spp. and *Paragonimus* spp.) (https://www.who.int/images/default-source/maps/ferg2010_trematode_a3_20240610.png) and (C) Aflatoxin (https://www.who.int/images/default-source/maps/ferg2010_aflatoxin_a3_20240610.png) (Geneva: World Health Organization; [2024]. License: CC BY-NC-SA 3.0 IGO). Republished from World Health Organization, Geneva under a CC BY license, with permission from Ian Coltart, Manager copyright; World Health Organization, Geneva, original copyright 2024. Data are based on WHO Foodborne Disease Burden Epidemiology Reference Group (FERG) 2007–2015 median estimates for 2010 [1].

While diarrhoeal disease agents were the leading cause of FBD burden in most global subregions (Table 1), specific agents of highest importance may vary by region and therefore the most effective point of intervention varies regionally, as indicated by three hazard-specific examples in Fig 1, indicating the differences between a globally ubiquitous hazard (*Salmonella*) and different regional hazards (intestinal flukes and aflatoxins).

For example, enteropathogenic *E. coli*, enterotoxigenic *E. col*i, and *Vibrio cholerae* were important causes of FBD in LMICs, whereas *Campylobacter* spp. was an important cause of FBD in high-income subregions [24]. Other foodborne agents are even more localized. The burden of aflatoxin is high in sub-regions of Africa, the Western Pacific, and SEA (Table 1), while *T. solium* is important in LMICs where pork is consumed (SSA, SEA and South America) and *Toxoplasma gondii* has a greater impact in Latin America, despite being a global issue [20]. In addition, the importance of specific foods as transmission routes vary by pathogen and the intake of different foods varies regional around the world. While global burden estimates convey a picture of the overall scope of the FBD health effects, these regional differences highlight the importance of regional, sub-regional and national incidence and burden estimates to prioritize country-level intervention programs aimed at reducing the burden of FBD [29].

## The economic imperative

FBDs have a significant economic impact globally. Even in those high-income countries (HICs) where FBD is comparatively well-controlled when compared with most LMICs, the economic impact remains meaningful. Indirect costs of FBD are typically poorly measured but can arise through impacts on nutritional status, child development, and food security initiatives, particularly in low-income countries (LICs) [54].

Food safety, phytosanitary, and animal health performance are crucial for LMICs to access high-value export markets. Total food exports from LMICs increased from USD 113 billion to USD 475 billion between 2001 and 2016. High value perishable food exports increased four-fold to USD 220 billion in 2016, 30% of which represented increased exports from LMICs [55]. Compliance with importing countries’ food safety standards grants market entry. Capacity to comply with food safety requirements is improving because food safety failures disrupt domestic and international markets, resulting in changes in management and market structure. Understanding the direct and indirect economic effects of FBD can promote interventions to address the socioeconomic disparities that arise from FBD (Box 1).

**Box 1. The socioeconomic burden of opisthorchiasis in Thailand.**

Opisthorchiasis caused by *Opisthorchis viverrini* is highly endemic in the Mekong River basin [56], and is associated with cholangiocarcinoma (CCA) [57]. Thailand has the highest reported incidence of CCA in the world with over 20,000 deaths a year [58] and DALYs of 216,530 [59]. The DALYs attributable to liver fluke infection were estimated between 70,745 and 138,221 (33%-64% of total DALYs due to CCA) [59] which is higher than those previously reported [20,60]. Liver fluke is largely a chronic asymptomatic infection and clinically CCA may arise several years after infection, hence the burden of disease is underestimated. Northeast Thailand is the poorest region affected by the liver fluke and CCA. Qualitative socioeconomic data have defined the financial burden on the family and community [58]. Most victims of CCA are small-scale farmers, primary school educated, and impoverished. Mortality occurs most predominantly in males aged 40–65 years, with children or grandchildren to support. Costs include pre-mortality cost to the family (e.g., paying for transport and accommodation to the hospital) and the costs for diagnosis, treatment and post-treatment palliative care that are covered by the Thai Universal Health Coverage scheme. The main costs, however, are likely to be post-mortality with loss of income and potentially the loss of a major contributor to farm work [58].

### Direct costs

The World Bank used WHO FBD estimates to calculate associated productivity losses, i.e., impact on time use. These cost LMICs USD 95.2 billion in 2016 [54]. This figure only captures labour market impacts of deaths and illnesses, excluding FBD medical treatment costs and financial impacts on quality of life. Productivity loss from FBD, as a percent of total gross national income, falls most heavily on LICs. Some HICs maintain national estimates of FBD costs, using more complete economic measures. These show that even where FBD is best controlled, the economic impact is significant (Table 1). The HIC estimates still underestimate the costs of chronic outcomes from FBD due to limited primary epidemiological and economic research [61]. Timing of analyses, currency fluctuations and analytical methods vary among HICs thus estimates are not directly comparable, but the magnitudes are illustrative of the economic cost FBD imposed (Table 1).

### Indirect costs

The effect of FBD on nutritional status and child development is a real but poorly measured impact on economic development [27,54], and weakens progress in LMICs towards sustainable development goals (SDGs) [54]. Repeated episodes of gastrointestinal illness or even asymptomatic infections with enteric pathogens, and aflatoxin exposures in LMICs has been associated with early childhood stunting [62–64], Nutrient malabsorption associated with diarrhoea reduces productivity [27], impairing physical and mental development throughout these individuals’ lifetimes [26,28,65]. Younger children are at enhanced risks from foodborne chemical exposures. In LMICs the economic burden of intellectual disability in children below seven years of age caused by lead exposure is an estimated USD 1 trillion [23,66].

Severe failures can erode consumer trust in food safety management, causing significant impacts and long-term changes in demand for particular foods or suppliers [55]. For example, a major outbreak of Shiga-toxigenic enteroaggregative *E. coli* (ST-EAEC) O104:H4 in Germany in 2011, initially erroneously linked to imported Spanish cucumbers, led to a significant drop in the consumption and export of Spanish vegetables in Europe. The error resulted in the European Union paying approximately 220 million Euros in compensation for income losses [18].

### Economic development may increase foodborne disease

As economies develop, diets shift from heavily carbohydrate-based to foods that carry increased risk of microbial contamination including increased fresh and perishable foodstuffs [55,67]. Food safety cycles through traditional diets to transitioning, modernizing, to a postmodern stage with improved safety at higher levels of economic development [54]. LMIC per capita consumption of meat, fruits and vegetables is expected to grow by 24% to 25% by 2050 [54,68]. Based on WHO data, experts estimated animal source foods contributed 35% of FBD globally in 2010 [69]. Longer and more complex supply chains raise food safety challenges, from increased storage and transportation times, to mixing of foods, expanding opportunities for cross-contamination in the food chain [34]. FBD levels are likely to rise as LICs develop, with lagging capacity to manage food safety, and then decrease as countries progress economically [54]. Improved governance, enhanced food safety capacity, national FBD surveillance and BoD estimates can ease the economic impact [29].

## The climate and planetary boundaries imperative

**Fig 2:**
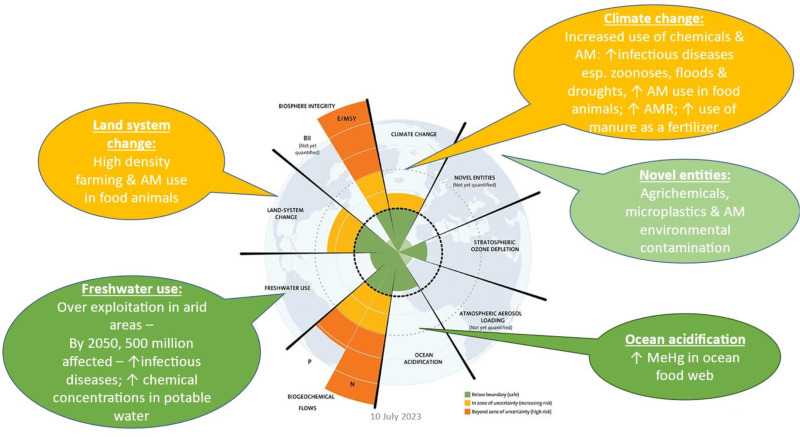
Estimates of how the different control variables for planetary boundaries have changed from 1950 to present. The green shaded polygon represents the safe operating space. Adapted from: Steffen et al 2015, https://www.stockholmresilience.org/research/planetary-boundaries.html, reproduced under license [70]. AM – Antimicrobials, AMR – Antimicrobial resistance, BII – Functional diversity, E/MSY – Genetic diversity, Novel entities – organic pollutants, radioactive materials, nanomaterials, microplastics, N – nitrogen, P – phosphorus.

Planetary boundaries represent nine quantitative processes that are intrinsically related to the stability and resilience of the Earth ecosystem [70,71], dysregulation of which may impact FBD (Fig 2). Exceeding certain planetary boundaries will directly affect food security, increasing FBD risk factors, such as those associated with climate change through extreme weather events, via temperature fluctuations, excessive rain, more frequent droughts [72,73], and increased environmental contamination with chemicals such as dioxins [70,71,74]. Indirectly, socio-ecological disruptions may cause food insecurity [75–77], destruction of agricultural land [71,78,79], contamination of freshwater sources for domestic and agricultural use, land system changes and losses due to urbanisation and rising sea levels, expanded geographical ranges of foodborne hazards, shifting infectious disease patterns including zoonotic diseases [47], and increased exposures to foods contaminated with chemicals and toxins [70,71,74,80].

### Food production changes and food security

The negative impacts of food security and food safety are mutually reinforcing [81], particularly in LMICs. Climate-related natural disasters lessen quality and quantity of crops needed to feed the world’s eight billion inhabitants, potentiating food insecurity, while compounding the current stressors on planetary boundaries [72,73,82]. If global mean temperatures rise 4°C or more, this could occur by 2050 [83]. Loss of biodiversity, including pollinators, will affect dietary composition [71], reducing fruit and vegetable consumption, with per-person reductions of 3.2% in global food availability, fostering the consumption of unsafe foods [84].

Threats posed by plant and animal diseases on food safety could be exacerbated by climate change (Table 1) [85]. Higher doses or increased use of more toxic plant pesticides and increased use of antimicrobials in animal husbandry may fuel the emergence of resistant strains of crop-destroying insects and pathogens, while increasing human exposures to harmful chemical compounds, antimicrobial residues, and drug-resistant foodborne pathogens [46,86–89]. Heavier rainfall increases quantities of heavy metals leaching into water systems, compounding toxic exposures through irrigation water [90]. Warming ocean temperatures are expanding the range of foodborne marine pathogens, and the planetary boundaries for green water, terrestrial precipitation, evaporation and soil moisture have already been transgressed [80]. Droughts will increase usage of water contaminated with sewage and animal faeces for irrigation [80,90], including for crops that are consumed raw, fostering exposure to faecal-orally transmitted pathogens (Table 1) [46,88].

Climate-related changes in diet and bodyweight, due to undernutrition or the consumption of cheap high calorie foods, are expected to result in 529,000 (95% CI 314,000–736,000) additional deaths in 2050, particularly in LMICs in Africa and SEA [84] These countries will bear the largest diminution in food availability, increasing deaths from malnutrition [82,84] and exposure to FBD due to consumption of unsafe foods [78,79]. Weak regulatory capability and governance to enforce and strengthen food safety and nutritional standards may potentially exacerbate these effects [91]. The flooding in Pakistan in 2022 illustrates what is to come (Box 2).

**Box 2. Planetary boundaries, food security, food safety and flooding in Pakistan.**

Pakistan ranks in the top 10 countries as being high-risk for climate-related events, with an average of 143 climate-related events annually between 1995 and 2014 [92] The flooding in Pakistan in 2022 highlighted the vulnerability of LMICs to the effects of failing planetary boundaries. A combination of excessive heat and associated glacial melt, and above average rainfall, with some areas experiencing up to five times the 30-year average [92,93], left two-thirds of the country under water and displacing 33 million people in 2022 [2]. Outbreaks of diarrhoeal disease associated with the floods, compounded by damage to healthcare centres, resulted in additional mortality [94].

Earlier floods in Pakistan in 2010 displaced 20 million people, submerging 20% of the country, creating economic and food insecurity in households [95]. Despite the distribution of food aid, 65% of households were unable to meet household needs up to six months later, reducing dietary quality by 75% [95]. Bokhari et al found diarrhoea due to *Escherichia coli* (DEC) in 57% of diarrhoeal specimens from cases during the 2010 floods, compared with 13% in the sporadic controls. The most frequent pathotypes were enteropathogenic *E. coli* (EPEC) (29%) and enterotoxigenic *E. coli* 29 (ETEC) (25%) [96]. A significant percentage, 36% of EPEC and 30% of ETEC, has been established as being foodborne [24].

Flooding is equally associated with excessive leaching of heavy metals from the soil [90]. While the toxicity of heavy metals is well-described, that of rare earth elements (REEs) has only recently been recognised [97–99]. The mountainous areas of northern Pakistan, home to some of the world’s largest glaciers, is rich in certain heavy metals and REEs [100,101]. Anthropogenic activities have resulted in excess heavy metals in drinking water in Pakistan [102]. Flooding of the agricultural land [93] risks foodborne exposure through increased concentrations of these compounds in crops, the health impact of which may not be measurable for years to come.

Sohail et al examined the persistence of organic pollutants along the course of the Indus River in Pakistan. They found that dioxin-like polychlorinated biphenyls (DLPCBs) were highest in the Indus Plain urban areas [103]. Higher levels of dioxins in farmed produce have been associated with exposure to industrial settlements, following groundwater and fresh water contamination [52,104]. Flooding of the Indus Plain agricultural areas has risked contamination by DLPCBs, potentially contaminating livestock and agricultural produce [105,106]. While their study focused predominantly on the presence of DLPCBs in dust, Sohail et al highlighted excessive risk of DLPBCs to children, particularly through ingestion [103].

### Emerging and re-emerging foodborne hazards

Emerging and re-emerging food risks can be unanticipated occurrences, due to accidental or natural contamination of foods, or alternatively deliberate contamination events [107]. These may be predicted through changes in drivers and enablers within influential sectors surrounding the food supply chain [107]. Driving forces include, *inter alia*, accelerating climate change, economic, environmental, and geopolitical forces and demographic changes, new food sources and production methods, resource shortages, increased complexity and extent of food supply chains, changes in water security, and limited political will and poor governance, both critical for resource allocation and food safety policy prioritization [107]. Altered precipitation, temperature, and extreme weather events are the three principal climate change factors leading to introduction of enteric pathogens and food contamination [89]. Marked seasonal variability has been associated with bacterial enteric infections and aflatoxin contamination, indicative of direct effects of climate change and warmer weather [45,49,108]. FBD incidence is expected to increase due to a warmer climate (Table 1).

Emerging food risks may be pre-existing but identified recently due to improved surveillance and detection techniques or an unintended consequence of control measures [107]. Contemporary evidence suggests increasingly common chronic diseases may be associated with foodborne exposure to certain chemicals, such as non-insulin dependent diabetes mellitus which has been linked to some high molecular wight (HMW) phthalates used in food packaging [109,110]. Increases of over 100% in certain HMW phthalate metabolites in urinary specimens were detected in a national survey in the United States between 2001 and 2010 [111]. Amongst other diseases, Parkinson’s disease has been significantly associated with trichloroethylene [112], an ubiquitous solvent that has been used in the production of decaffeinated coffee, beer, extraction of vegetable oils and preparation of flavouring extracts [113].

Global warming and geoclimatic variations impact the epidemiology of zoonoses. Alterations in the interactions between animal hosts, vectors, climate conditions, pathogens and transmission dynamics will affect susceptible human populations [114]. Changes in animal husbandry, increasing transmission of zoonotic pathogens among animals, or novel weather patterns altering environmental survival of zoonotic pathogens may equally increase FBD incidence (Table 1) [89]. Regular review of BoD data will monitor changes in foodborne hazards [1,29], enabling pre-emptive action and mitigating climate-related health and socioeconomic effects [89].

## The governance imperative

Good governance requires a clear quantitative picture for managing food safety and recognition of the limitations of surveillance data in providing a clear quantitative picture of FBD burdens, while integrating the health, economic and planetary boundary imperatives. Although surveillance data can provide useful information on disease and exposure trends, it is not available for all foodborne hazards or in all settings, particularly in resource-restricted countries [1], which may be incomplete due to under-diagnosis and underreporting.

Differences in food handling, contamination, consumption patterns and FBD globally necessitate national FBD burden estimates to rank the relative impacts on human health, prioritise food safety interventions, and define the public health and economic impact of FBD to reduce FBD burdens. National BoD data can improve understanding of the extent of FBD. BoD data should adjust for underreporting and under-diagnosis and measure the link between FBD and the associated sequelae [29]. By ranking hazards according to risk, interventions can be prioritized to most impact FBD-associated disability-adjusted life years (DALYs) [1,29]. The WHO estimates highlighted a critical data need for estimating the BoD in some of the world’s higher middle income and most populous countries, such as China and Russia [1]. LMICs in SSA, south Asia and SEA had the highest FBD burdens [1], but again scarcity of data increased uncertainty around national, regional, and global estimates.

Well-conducted studies on the extent and cause of FBD, paired with an effective and iterative FBD surveillance system that supports hazard-specific interventions, result in measurable progress in population health and economic stability. The robust FBD surveillance system in New Zealand supported an active control programme for one of the highest campylobacteriosis burdens globally, reducing disease notifications from 2005 to 2008, from 400 to 150 cases per 100,000 population [115]. Defining *Salmonella* burdens in chicken and pigs provided the foundation to reduce the pathogen’s prevalence in Danish flocks and herds, saving USD 25.5 million for the Danish public [116].

Although the health benefits may not be immediately quantifiable, a decline in measurable foodborne hazards following legislative interventions is indicative of the potential of FBD BoD studies to guide evidence-based interventions to reduce exposure. Health risks associated with antibiotic residues in food animals include bacterial resistance, hypersensitivity reactions, toxicity, teratogenicity and cancer [117]. Abolishing antimicrobials as growth promoters in food animals resulted in significant declines in resistant bacteria isolated from broilers and pigs in Denmark by up to a factor of 12 between 1995 and 2000 [118]. Antibiotics are now abolished in the European Union as growth promoters [117]. The WHO has issued guidelines to limit the use of medically important antimicrobials in food-producing animals [119].

Humans are predominantly exposed to dioxins through the food supply, particularly animal fats and dairy products [51]. Countries that measure dioxin levels in food, recalling foods exceeding these, have measured temporal declines in dioxin levels in meat, dairy and shellfish, and resultant human exposures [104–106]. In Japan dioxin levels in human breast milk decreased from 21pg toxic equivalence (TEQ) per gram of fat 1998–7pg TEQ/g fat between 1998 and 2014 [120].

Despite the paucity of data, persistent food safety failures in LMICs can result in long-term structural changes in demand to promote improved governance and changes in market organization. In West Africa for example, increased demand for imported infant formula [121], was supported by strengthened oversight and quality control of vertical coordination of milk markets in China, followed the 2008 melamine milk contamination scandal [122,123].

Improved understanding of the combined effects of diseases that are common to humans and animals is needed [124]. Zoonotic diseases that sicken food animals and poultry, doubly affect food security by decreasing supplies and increasing FBD exposures. National programmes to measure the dual burden of zoonotic diseases, including FBDs, on humans and animals, have been created. The Global Burden of Animal Diseases (GBADs) has defined the economic importance of animals, documenting the human cost of poor animal health and welfare, including veterinary support, which can be measured at an economically relevant level zDALY (Box 3) [125,126].

**Box 3. Quantifying the societal burden of foodborne pathogens with the zDALY at a national level as an indicator of human health and socioeconomic effect of zoonotic foodborne disease [126].**

The zDALY is essentially the DALY component due to human disease calculated in a standard manner, added an animal life equivalent (ALE) which is the time trade-off in human life years for animal losses (DALY+ALE = zDALY) [126]. ALE is calculated as the time taken to earn the income to replace the animal loss analogous to methods used for estimating disability weights for non-fatal human disease. Other methods such as preparedness to pay, either in money (converted to time by labour costs) or directly in time, with pairwise comparisons with human diseases, are possible. zDALYs are core to understanding excessive disease burdens in LMICs. In Paraguay the burden of zoonoses used the zDALY approach [127]. The study estimated that the annual burden of DALYs was approximately 19,000. Addition of the ALE to this total demonstrated that the burden of zDALYs was approximately 62,000 [127]. In Morocco, DALYs due to cystic echinococcosis were estimated at 0.5 years per 100,000 population, compared with zDALYs of 55 years per 100,000 population [128].

Halving the green-house gas emissions and keeping the global temperature increases <1.5°C was the global goal set forth by the Paris Agreement at the 21^st^ Conference of Parties (COP21) in 2015 [129]. Recent COP meetings were lost opportunities for governments to align monitoring national burdens of FBD and the impact of climate change as key measurements for planetary health [130,131], despite acknowledging the impact of climate change on food security [131]. Due to the excessive DALYs, mortality, and zDALY and socioeconomic costs, good governance requires a national policy to define FBD burdens, ranking the most significant national hazards associated with these planetary stressors [1], and defining strategies that will improve the multiple consequences of FBD.

## Conclusion

This review highlighted four global and national imperatives for strengthening FBD disease surveillance and burden estimation at a national level to formulate risk ranking of different foodborne hazards. Targeted programmes to maintain international standards safeguarding consumers’ health and international trade will also protect local populations and national economies. Transparency regarding foodborne BoD will promote defined interventions according to the calculated risks, enhancing national reputation and trade for food-producing countries. Detailed methodologies to achieve national BoD estimates are available for countries to embark on these processes [29].

The content of this review was constrained by an absence of data for many of the foodborne hazards, including BoD, economic costs and weaknesses associated with modelling for potentiation or mitigation of FBD burdens at a national, and sometimes global level. Hence many examples included here have been guided by limited numbers of national studies or data models that may not fully represent the extent of FBD.

Sustained BoD programmes complimented by surveillance for FBD can measure the success of these impacts, augment prospects for the early detection of new hazards and improve rapid response to FBD outbreaks. Such programmes will create opportunities enhancing education to prevent FBD in the agriculture sector, among food producers, in the home and the informal sector [132–135]. Legislative interventions, such as registration of street food vendors, can counterbalance the effects associated with their limited training [133,134]. Equally, creation of a robust programme encompassing FBD surveillance and reporting, monitoring national BoD estimates, will provide a further bastion against threats to the planetary boundaries. Countries effectively addressing national FBD burdens can ease over-encumbered healthcare systems, using evidenced-based interventions, to improve health and socioeconomic circumstances of the most vulnerable.
